# Retrospective analysis of clinical phenotype and prognosis of hypertrophic cardiomyopathy complicated with hypertension

**DOI:** 10.1038/s41598-019-57230-z

**Published:** 2020-01-15

**Authors:** Qin Luo, Jin Chen, Tianhua Zhang, Xiaoyu Tang, Bilian Yu

**Affiliations:** Department of Cardiovascular medicine, The Second Xiangya Hospital, Central South University, Changsha, Hunan P.R. China

**Keywords:** Cardiac hypertrophy, Hypertension

## Abstract

We here studied the clinical features, cardiac structure, and functional changes and prognosis of hypertrophic cardiomyopathy (HCM) patients with hypertension (HTN). A total of 90 HCM patients with HTN and 172 patients without HTN were divided into a hypertensive group and non-hypertensive group. The clinical characteristics, cardiac structure and function, and prognosis of the two groups were compared. Our study found that HCM patients with HTN had fewer syncope events in their medical histories (8% vs. 22%, *P* < 0.01) and sudden deaths in the family (3% vs. 10%, *P* < 0.05). The prevalence of apical hypertrophy (18% vs. 7%, *P* < 0.01) and midventricular obstruction (26% vs. 15%, *P* < 0.05) was higher in the HTN group. Besides, simple HCM patients had more pathogenic gene mutations, while those with HTN were more likely to have mutations of uncertain clinical significance (64% vs. 24%, *P* < 0.05). Evaluation of 5-year survival rate showed a trend for a worse prognosis in HCM patients with HTN, but the results were not statistically insignificant (*P* = 0.065). In conclusion, we found that the clinical phenotypes of HCM patients with HTN differed from those of patients with HCM alone, suggesting that HTN may play a pathogenic role in the pathogenesis of hypertensive hypertrophic cardiomyopathy patients.

## Introduction

Hypertrophic cardiomyopathy (HCM) is a heterogeneous monogenic heart disease of unknown origin characterized by asymmetric hypertrophy of the ventricular wall^1^. Mutations in genes encoding the sarcomere or sarcomere-associated proteins lead to the left ventricular hypertrophy (LVH) in HCM. The pattern and the distribution of LVH in HCM are variable. Hypertrophy can be isolated to the inter-ventricular septum, left ventricular free wall, apex, anterolateral wall, papillary muscles, and right ventricle, but concentric hypertrophy is rarely described^2^. The diagnosis of HCM should exclude other causes of myocardial hypertrophy, such as hypertension (HTN), rheumatic heart disease, congenital heart disease, or myocardial infiltrative diseases such as amyloidosis or glycogen storage disease.

HTN can also cause myocardial hypertrophy, which is characterized by concentric hypertrophy. Hemodynamic overloading and the subsequent proliferation of sarcomere protein may be the main causes of myocardial hypertrophy in patients with HTN^3,4^. The main differences between HTN with concentric LVH and HCM include systolic anterior motion (SAM), early diastolic time intervals, and differences in long-axis systolic and diastolic left ventricular functions^3^.

However, co-occurrence of HTN and HCM is not uncommon in clinical practice. In 1985, Topol *et al*.^5^ found some hypertension patients characterized as concentric hypertrophy, abnormal diastolic function, and hyperdynamic left ventricular contraction and put forward the concept of “hypertensive hypertrophic cardiomyopathy” (HHCM). However, considering HTN was not the primary cause of the cardiomyopathy, the “2011 ACCF/AHA Guideline for the Diagnosis and Treatment of Hypertrophic Cardiomyopathy” stated that the patients with a history of HTN and characterized as a diagnostic sarcomere mutation or marked ventricular wall thickness >25 mm, LVOT obstruction, or both induced by systolic anterior motion (SAM), can be classified as HCM with HTN^6^. However, it is not clear whether HTN plays a role in the development of disease in these patients. Recently, it has been noted that the myocardial hypertrophy of some patients with HTN is not typical hypertensive concentric hypertrophy, and their blood pressure was found to decrease when the cardiac hypertrophy-induced obstruction appeared, which raises the possibility that HTN might act as an important pathogenic or auxiliary factor in the development of HCM. Herein, we tried to study the clinical features, cardiac structure, and functional changes and prognosis of HCM patients with HTN, so providing reference data for the clinical diagnosis and treatment of HCM.

## Methods

### Patients

The study participants were 262 patients diagnosed with HCM in the department of cardiovascular medicine the Second Xiangya Hospital between 2014 and 2018. The patients were divided into two groups based on whether they had HTN. There were 90 patients with HTN and 172 without. Among them, there were 28 cases of apical hypertrophy (APH) and 49 cases of midventricular obstruction (MVO). According to the presence or absence of HTN, the patients were further divided into HTN group and non-HTN group. Thus, in the APH group, there were 15 participants with HTN and 13 without; in the MVO HCM group, there were 23 participants with HTN and 26 without. The age at diagnosis, symptoms, complications, electrocardiogram, echocardiographic parameters, gene sequencing, and prognosis were compared between the two groups of patients.

All data were sourced from the electronic medical record systems or collected via detailed telephone follow-up. The study was approved by the Institutional Review Board of the Second Xiangya Hospital.

### Diagnostic criteria

The diagnosis of HCM was based on an unexplained LV wall thickness of ≥15 mm or 13 mm in the presence of a first-degree family member affected by HCM^7^. Patients with myocardial hypertrophy secondary to amyloidosis, aortic stenosis, or hypothyroidism were excluded. HTN was diagnosed when the resting systolic blood pressure was >140 mmHg or the diastolic blood pressure was >90 mmHg. MVO was diagnosed when the peak instantaneous midventricular gradient was estimated to be ≥30 mmHg as assessed by left ventriculography^8^. The diagnostic criteria for APH included asymmetric left ventricular hypertrophy that was confined predominantly to the left ventricular apical region, along with an apical wall thickness ≥15 mm^9^.

### Echocardiography

Echocardiographic images and data were collected according to the 2003 guidelines of the US Society of Echocardiography.

### Follow-up

Patients were followed up via telephone interviews. The final follow-up was performed on March 5, 2019.

### Genome-wide sequencing

Written consent for blood collection and genome-wide sequencing was obtained from each patients. A Trio-WES strategy was applied to identify the causal variants from all the patients. Briefly, genomic DNA was extracted, hybridized and enriched according to the established protocols. The sequencing data were aligned to the human reference genome (hg19/GRCh37) and PCR duplicates were removed by using Picard v1.57 (http://picard.sourceforge.net/). Verita Trekker® Variants Detection Systemby Berry Genomics and GATK (https://software.broadinstitute.org/gatk/) were employed for variant calling. ANNOVAR^10^ and the Enliven® Variants Annotation Interpretation System authorized by Berry (BerryGenomics, China) were used for variant annotation and interpretation. According to the American College of Medical Genetics and Genomics (ACMG) guidelines for interpretation of genetic variants^11^, the variants were classified to five categories: “pathogenic”, “likely pathogenic”, “uncertain significance”, “likely benign” and “benign”. The identified mutations among all family members were validated by Sanger sequencing.

### Statistical analysis

SPSS 21.0 statistical software was used for statistical analysis. Categorical variables are here reported in frequencies and percentages, and the difference between groups was tested with the *X*^2^ test. Continuous variables are expressed as mean and standard deviation, and the difference between groups was tested with the *t* test method. Statistical significance was at *P* ≤ 0.05. Survival analysis is here described by using the Kaplan-Meier survival analysis method.

### Ethical approval and informed consent

All procedures performed in studies involving human participants were in accordance with the ethical standards of the institutional research committee (Ethics Committee of Second Xiangya Hospital) and with the 1964 Helsinki declaration and its later amendments or comparable ethical standards. Written informed consent was obtained for each patients.

## Results

### Baseline characteristics of all HCM patients

A total of 262 patients were enrolled. There were 90 HCM patients with HTN and 172 without. Baseline characteristics of all HCM patients are shown in Table 1. In the entire population, patients with HTN were older at diagnosis (55 ± 12.5 vs. 47 ± 16.2, *P* < 0.001) and had a greater prevalence of hyperlipidemia (38% vs. 22%, *P* < 0.01), transient ischemic attack (8% vs. 2%, *P* < 0.05), chronic obstructive pulmonary disease (7% vs. 2%, *P* < 0.05), and chronic renal failure (5% vs. 0.6%, *P* < 0.05). Importantly, HCM patients with HTN had a significantly lower prevalence of syncope (8% vs. 22%, *P* < 0.01) and sudden death (3% vs. 10%, *P* < 0.05). Arrhythmia is one of the main causes of syncope in HCM patients. However, we found no significant differences in the incidence of ventricular tachycardia, atrial fibrillation, ICD, or pacemaker implantation. The family history of HCM showed a trend for a decrease in HCM patients with HTN, but the changes were not statistically significant (8% vs. 16%, *P* > 0.05).

**Table 1:** Baseline characteristics of HCM patients.

	No HTN (n = 172)	HTN (n = 90)	P-value
**Demographics**			
Age at HCM diagnosis	47 ± 16.2	55 ± 12.5	<0.001
Male gender	101 (59%)	61 (68%)	>0.05
**Medical history and cardiovascular risk factors**			
Hyperlipidaemia	35 (22%)	31 (38%)	<0.01
Current smoking	35 (22%)	21 (26%)	>0.1
Coronary artery disease	35 (22%)	27 (33%)	>0.05
TIA	3 (2%)	7 (8%)	<0.05
Chronic obstruction pulmonary disease	3 (2%)	6 (7%)	<0.05
Chronic renal failure	1 (0.6%)	4 (5%)	<0.05
History of VT	21 (12%)	8 (9%)	>0.05
**Clinical features**			
Angina pectoris	76 (44%)	43 (48%)	>0.9
Personal history syncope	37 (22%)	7 (8%)	<0.01
Atrial fibrillation	50 (29%)	31 (34%)	>0.05
Family history HCM	27 (16%)	7 (8%)	>0.05
Family history sudden cardiac death	18 (10%)	3 (3%)	<0.05
**Device implantation**			
Permanent pacemaker	12 (7%)	8 (9%)	>0.05
Implanted defibrillator	6 (3%)	1 (1%)	>0.05
Death cases in 5-year follow-up	7 (4%)	8 (9%)	>0.05
**Causes of death**			
Cardiac related death^a^	5 (71%)	3 (38%)	>0.05
Non-cardiac related death^b^	2 (29%)	5 (62%)	

Categorical parameters are presented as n(%). Continuous variables are expressed as mean and standard deviation. HCM, hypertrophic cardiomyopathy; TIA, transient ischaemic attack; VT, ventricular tachycardia. ^a^Acute heart failure, arrhythmia, myocardial ischemia. ^b^Infection, stroke, chronic renal failure and chronic obstruction pulmonary disease.

### Electrocardiographic and echocardiographic features of all HCM patients

The ECG and echo-Doppler characteristics of all HCM patients with HTN are shown in Table 2 and compared to those of patients without hypertension. Patients with HTN had a higher prevalence of P-mitrale (35% vs. 16%, *P* < 0.01) and enlarged LV diastolic dimension (47 ± 12.3 vs. 44 ± 7.4, *P* = 0.019). There were no differences in the interventricular septum, left atrial diameter, LV ejection fraction, or diastolic function between HCM patients with and without HTN. HCM patients did not differ in the prevalence of thickest basal part, LVOT obstruction, mitral valve regurgitation, or significant pulmonary arterial hypertension. APH and MVO were more common among patients with HTN (APH: 7% vs. 18%, *P* < 0.01, MVO: 15% vs. 26%, *P* < 0.05). Besides, the pressure gradient of LVOT was smaller in patients with HTN (70 ± 5.1 vs. 52 ± 6.2, *P* < 0.05). In terms of ECG features, although patients with HTN had a higher prevalence of enlarged LV diastolic dimension, the high voltage of LV showed a trend toward increase in HCM patients with HTN. Heart conduction blockage, baseline PR, and QRS intervals did not differ significantly between groups.

**Table 2:** Electrocardiographic and echocardiographic indices of HCM patients.

	No HTN (n = 172)	HTN (n = 90)	P-value
**Electrocardiography**			
LV high voltage	95 (60%)	57 (70%)	>0.05
Sinus rhythm	116 (73%)	63 (78%)	>0.05
P-mitrale	26 (16%)	28 (35%)	<0.01
PR duration (s)	159 ± 31.5	166 ± 30.9	0.18
QRS duration (s)	105 ± 23.1	103 ± 23.5	0.55
Right bundle branch block	15 (9%)	4 (5%)	>0.05
Left bundle branch block	8 (5%)	4 (5%)	>0.05
Left anterior fascicular block	20 (12%)	7(9%)	>0.05
Atrioventricular nodal block >1st degree	7 (4%)	4 (5%)	>0.05
**Echocardiography**			
Interventricular septum (mm)	19 ± 5.3	18 ± 5.8	0.29
Thickest basal part	18 (10%)	10(11%)	>0.05
LV ejection fraction (%)	59 ± 9.3	60 ± 8.1	0.22
Left atrial diameter (mm)	41 ± 6.7	42 ± 5.6	0.34
LV diastolic dimension (mm)	44 ± 7.4	47 ± 12.3	0.019
Mitral regurgitation >mild	48 (28%)	32 (36%)	>0.05
SPAP ≥ 45 mmHg	17 (10%)	9 (10%)	>0.05
Mid ventricular obstruction	26 (15%)	23 (26%)	<0.05
LVOT obstruction	37(22%)	18 (20%)	>0.05
Apical HCM	12 (7%)	16 (18%)	<0.01
LVOT PG, mmHg	70 ± 5.1	52 ± 6.2	<0.05
Diastolic dysfunction - any grade	129 (81%)	65 (80%)	>0.05

Categorical parameters are presented as n(%). Continuous variables are expressed as mean and standard deviation. LV, left ventricular; SPAP, estimated systolic pulmonary arterial pressure; LVOT, LV outflow tract; PG, pressure gradient; HCM, hypertrophic cardiomyopathy.

### Baseline characteristics and electrocardiographic and echocardiographic features of apical HCM and MVO HCM patients

In the APH group (Table 3), there was no statistical difference between patients with or without HTN because of a limited sample size. In the APH group (Table 4), apical HCM patients with HTN had thicker interventricular septum (13 ± 2.8 vs. 11 ± 1.3, *P* = 0.014) and maximum left ventricular wall thickness (15 ± 4.4 vs. 11 ± 2.9, *P* = 0.021), but there was no significant difference in apical thickness. In the MVO group (Tables 5 and 6), there was no significant difference in baseline characteristics and ECG or echo-Doppler parameters between patients with and without HTN.

**Table 3:** Baseline characteristics of HCM patients with APH.

	APH		
	With HTN (n = 15)	Without HTN (n = 13)	P-value
**Demographics**			
Age at HCM diagnosis	54 ± 14.1	52 ± 15.6	0.756
**Medical history and cardiovascular risk factors**			
Hyperlipidaemia	4 (27%)	4 (31%)	>0.05
Current smoking	1 (7%)	4 (31%)	>0.05
CAD	4 (27%)	6 (46%)	>0.05
TIA	3 (20%)	0 (0%)	>0.05
Chronic renal failure	0 (0%)	0 (0%)	>0.05
History of VT	2 (13%)	1 (8%)	>0.05
**Clinical features**			
Angina pectoris	6 (40%)	4 (31%)	>0.05
Syncope	1 (7%)	0 (0%)	>0.05
Atrial fibrillation	6 (40%)	2 (15%)	>0.05
Family history HCM	1 (7%)	2 (15%)	>0.05
sudden cardiac death	0 (0%)	1 (8%)	>0.05

Categorical parameters are presented as n(%). Continuous variables are expressed as mean and standard deviation. HCM, hypertrophic cardiomyopathy; CAD, coronary artery disease; TIA, transient ischaemic attack; VT, ventricular tachycardia.

**Table 4:** Electrocardiographic and echocardiographic indices of HCM patients with APH.

	APH		
	With HTN (n = 15)	Without HTN (n = 13)	P-value
**Electrocardiography**			
LV high voltage	11 (73%)	10 (77%)	>0.05
Sinus rhythm	12 (80%)	11 (85%)	>0.05
P-mitrale	6 (40%)	2 (15%)	>0.05
PR duration (s)	151 ± 27.7	147 ± 20.2	0.697
QRS duration (s)	101 ± 22.0	89 ± 12.2	0.082
Right bundle branch block	0	0	>0.05
Left bundle branch block	0	0	>0.05
Left anterior fascicular block	0	0	>0.05
Atrioventricular nodal block > 1st degree	1 (7%)	0	>0.05
**Echocardiography**			
Interventricular septum (mm)	13 ± 2.8	11 ± 1.3	0.014
Thickest basal part	0	0	>0.05
MLVWT	15 ± 4.4	11 ± 2.9	0.021
LV ejection fraction (%)	60 ± 5	62 ± 6	0.574
Left atrial diameter (mm)	41 ± 4.5	35 ± 6.1	0.015
LV diastolic dimension (mm)	55 ± 26.2	44 ± 14.3	0.216
Apical thickness	16 ± 2.1	15 ± 2.0	0.066

Categorical parameters are presented as n(%). Continuous variables are expressed as mean and standard deviation. LV, left ventricular; MLVWT, maximum left ventricular wall thickness.

**Table 5:** Baseline characteristics of HCM patients with MVO.

	MVO HCM		
	With HTN (n = 23)	Without HTN (n = 26)	P-value
**Demographics**			
Age at HCM diagnosis	57 ± 9.1	46 ± 17.1	0.008
**Medical history and cardiovascular risk factors**			
Hyperlipidaemia	7 (30%)	4 (15%)	>0.05
Current smoking	5 (22%)	5 (21%)	>0.05
CAD	8 (35%)	9 (35%)	>0.05
TIA	2 (9%)	0 (0%)	>0.05
Chronic renal failure	0 (0%)	0 (0%)	>0.05
History of VT	1 (4%)	0 (0%)	>0.05
**Clinical features**			
Angina pectoris	14 (61%)	16 (62%)	>0.05
Syncope	3 (13%)	7 (27%)	>0.05
Atrial fibrillation	8 (35%)	5 (19%)	>0.05
Family history HCM	2 (9%)	7 (27%)	>0.05
sudden cardiac death	1 (4%)	4 (15%)	>0.05

Categorical parameters are presented as n(%). Continuous variables are expressed as mean and standard deviation. HCM, hypertrophic cardiomyopathy; CAD, coronary artery disease; TIA, transient ischaemic attack; VT, ventricular tachycardia.

**Table 6:** Electrocardiographic and echocardiographic indices of HCM patients with MVO.

	MVO HCM		
	With HTN (n = 23)	Without HTN (n = 26)	P-value
**Electrocardiography**			
LV high voltage	20 (87%)	21 (81%)	>0.05
Sinus rhythm	18 (78%)	23 (88%)	>0.05
P-mitrale	8 (35%)	5 (19%)	>0.05
PR duration (s)	158 ± 27.5	153 ± 20.0	0.517
QRS duration (s)	96 ± 12.7	101 ± 20.1	0.309
Right bundle branch block	0	2 (8%)	>0.05
Left bundle branch block	2 (9%)	2 (8%)	>0.05
Left anterior fascicular block	1 (4%)	5 (19%)	>0.05
Atrioventricular nodal block >1st degree	1 (4%)	0	>0.05
**Echocardiography**			
Interventricular septum (mm)	20 ± 6.2	19 ± 5.1	0.562
Thickest basal part	8 (35%)	7 (27%)	>0.05
MLVWT	22 ± 5.6	21 ± 5.1	0.72
LV ejection fraction (%)	63 ± 8.5	64 ± 5.9	0.587
Left atrial diameter (mm)	43 ± 6.2	42 ± 4.9	0.328
LV diastolic dimension (mm)	43 ± 6.0	41 ± 4.8	0.243

Categorical parameters are presented as n(%). Continuous variables are expressed as mean and standard deviation. LV, left ventricular; MLVWT, maximum left ventricular wall thickness.

### Genome-wide sequencing

Among 262 patients, 50 HCM patients underwent genome-wide sequencing (Tables 7 and 8). The results showed that simple HCM patients had more pathogenic gene mutations, while those with HTN were more likely to have mutations of uncertain clinical significance (64% vs. 24%, *P* < 0.05). As shown in Table 8, β-myosin heavy chain (*MYH7*) and cardiac myosin-binding protein C (*MYBPC3*) gene predominated in frequency in patients with HCM alone, in contrast, *MYBPC3* and Titin (*TTN*) gene account for most of the gene mutation in HCM patients with HTN.

**Table 7:** Genome-wide sequencing of partial HCM patients.

	No HTN (n = 33)	HTN (n = 17)	P-value
Pathogenic gene mutation	21 (64%)	4 (24%)	P < 0.05
Clinical significance is unclear	11 (33%)	11 (65%)	
No gene mutation	1 (3%)	2 (12%)

**Table 8:** Genome-wide sequencing data of partial HCM patients.

Gene	cases	mutation	Pathogenic cases	uncertain significance cases	HTN cases (Pathogenic/uncertain significance)	No HTN cases (Pathogenic/uncertain significance)
MYH7	13	c.428G > A, c.2011C > T, c.3382G > A, c.428G > A, c.4145G > A, c.1322G > A, c.4559G > T, c.2200C > G, c.4145G > A, c.1816G > A, c.4124A > G, c.3341G > A, c.1498G > C	7	6	2 (2/0)	11 (5/6)
MYBPC3	14	c.3041delT, c.3624delC, c.2522_2525Dup, c.1377delC, c.821 + 1G > A, c.3307C > T, c.3624delC, c.1591G > A, c.1377delC, c.3814 + 2T > G, c.1303C > T, c.1493delA, c.2551G > A, c.873del	10	4	3 (0/3)	11 (10/1)
TTN	9	c.12889 + 1G > T, c.74722C > T, c.95968C > T, c.90091 + 1G > A, c.92183C > T, c.77387G > A, c.70315C > T, c.19297G > A, c.72105_72107delTGT	1	8	5 (1/4)	4 (0/4)
TNNT2	3	c.856C > T, c.418C > T, c.304C > T	3	0	1(1/0)	2 (2/0)
MAP2K1	1	c.199G > A	1	0	0	1 (1/0)
MYPN	2	c.1840G > A, c.52G > A	0	2	0	2 (0/2)
TNNI3	2	c.460A > G, c.611G > A	0	2	0	2 (0/2)
MYH6	1	c.2353C > T	0	1	0	1 (0/1)
MYO6	1	c.702G > A	0	1	0	1 (0/1)
DTNA	1	c.1023C > G	0	1	1 (0/1)	0
JPH2	1	c.521C > G	0	1	0	1 (0/1)
GLA	1	c.1228A > C	0	1	1 (0/1)	0
MYH11	1	c.2323A > C	0	1	1 (0/1)	0
SYNE2	1	c.19975C > T	0	1	1 (0/1)	0
NODAL	1	c.358G > A	0	1	1 (0/1)	0
ILK	1	c.1357T > C	1	0	0	1 (1/0)
SERPINB4	1	c.154C > T	1	0	0	1 (1/0)
HCN4	1	c.229A > C	0	1	1 (1/0)	0
GTPBP3	1	c.575_576del	1	0	0	1 (1/0)

### Outcomes and mortality

**Figure 1:**
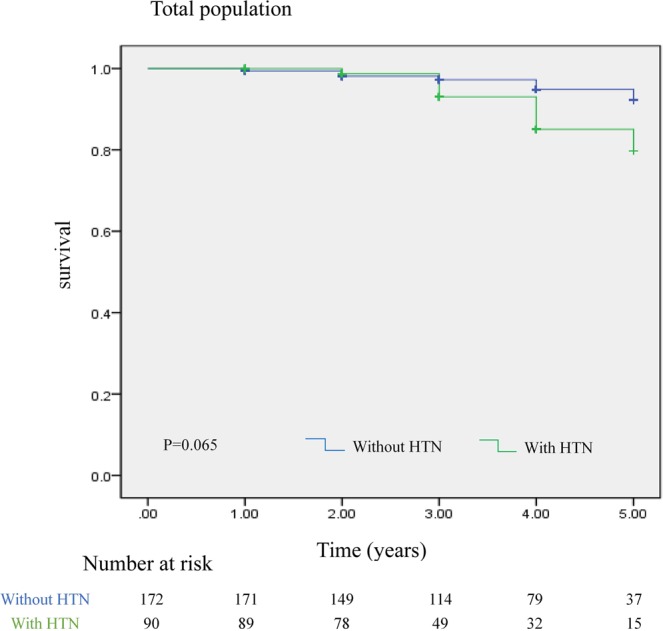
Kaplan-Meier estimates of HCM patients with and without HTN.

In this retrospective analysis, the longest follow-up lasted 5 years. A Kaplan-Meier plot (Fig. 1) here shows that the survival curves separate after the 3-year mark, favoring a better long-term survival in non-hypertensive patients. However, the results were not statistically significant (*P* = 0.065). We specified the cause of death in HCM patients with HTN in Table 1. Due to the small number of cases, it is not clear whether non-cardiac related death leads to the difference in prognosis between the two groups. However, there was a trend that the percentage of non-cardiac death was higher in HCM patients with HTN.

## Discussion

Our study analyzed two groups of HCM to examine the effect of coexisting HTN on the clinical features and prognosis of HCM. When compared with patients with HCM alone, the clinical features of HCM patients with HTN were predominated by older age, lower prevalence of syncope and sudden death, and higher prevalence of P-mitrale and enlarged LV diastolic dimension. Apical HCM and MVO were more common among HCM patients with HTN than in those without. There was no significant difference in systolic or diastolic function or in incidence of atrial fibrillation. The 5-year survival rate showed a trend for a worse prognosis in HCM patients with HTN, but the results were not statistically significant.

Aslasm *et al*.^12^ compared 122 patients with HCM plus HTN to 74 patients with HCM alone. Consistent with our studies, in terms of baseline characteristics, HCM patients with HTN were older at diagnosis than those with HCM alone, suggesting that their hypertrophic phenotype may occur later than that of patients with HCM alone. Unlike in the present study research, however, Aslasm *et al*. found no significant difference in electrocardiographic changes, echocardiographic indices, or the prevalence of such symptoms as chest pain, palpitation and syncope, or heart failure between two groups. Tarazi and Levy^13^ argued that the severity of hypertrophy often could not be related to the severity or duration of HTN. Investigators eventually concluded that HTN was not necessarily a factor for myocardial remodeling but may be an auxiliary factor^12^. However, our study found that the phenotype of myocardial hypertrophy in HCM patients with HTN was different from that in patients with HCM alone, suggesting that HTN not only may play an auxiliary role in HHCM patients but rather may be one of the pathogenic factors.

We here attempt to explain the causes of phenotypic differences from the perspective of etiology. The distribution of gene mutations in 31 patients with HHCM was examined at Massachusetts General Hospital in 2002, which was significantly different from the simple HCM^14^. There were no mutations in MYH7, TNNT, or TPM1 (0 vs. 45%) but mainly MYBPC3, TNNI, and a-MHC. In our study, the results of gene sequencing of 50 HCM patients in our hospital were collected (Table 7). We found that the pathogenic gene mutations tended to increase in simple HCM patients, while those with HTN were more likely to have mutations of unknown clinical significance. These findings seem to suggest that the gene distribution in HCM patients with HTN was different from that of patients with HCM alone. This may be why the clinical phenotypes of HCM patients with HTN differed from those of patients with HCM alone. We speculate that HCM patients with HTN have some form of genetic susceptibility, which eventually leads to myocardial hypertrophy on the basis of HTN. However, whether there are differences in gene mutations between two groups requires a larger sample size and remains to be further studied.

As shown in our study, APH and MVO are more prevalent in HCM patients who also have HTN. We then attempted to determine why APH and MVO phenotypes were more common in hypertensive patients. The relationship between HTN and these two special phenotypes is still unclear. Because of the mild degree of left ventricular obstruction in patients with MVO and APH^8,15^, which might not have a pronounced effect on arterial blood pressure, it is easier to detect HTN in HCM patients, indicating that there might be no causal relationship between HCM and these two phenotypes. However, in 1985, Koga *et al*.^16^ studied the acquired factors of apical hypertrophy by a relevancy analysis on HTN and APH and found that transient hypertension during daily activity was associated with apical hypertrophy. Harrison *et al*.^17^ reported 10 hypertensive patients with remarkable concentric left ventricular (LV) hypertrophy and MVO but no family history of HCM, indicating that the gross hypertrophy of papillary muscles and the interventricular septum induced by hypertension might lead to left ventricular obstruction. Thus, it is plausible that, under certain conditions, HTN itself can lead to APH and MVO rather than LVOT obstruction, and HTN might be a pathogenic factor, participating in the pathogenesis of HHCM.

Another difference between the two groups was the higher prevalence of syncope and sudden death in HCM patients without HTN. There are two main causes of syncope and sudden death in HCM patients: arrhythmia and a primary hemodynamic mechanism^18^. Arrhythmia includes paroxysmal atrial fibrillation, sustained ventricular tachycardia, and other tachyarrhythmia or bradyarrhythmia. It has been reported that patients with mutations in *MYH7* usually have higher rate of cardiac conduction disease, ventricular arrhythmia and sudden death^19^, thus, the higher frequency of *MYH7* mutation in HCM patients without HTN might attributed to the higher prevalence of syncope and sudden death. The hemodynamic mechanism includes LVOT and abnormal vascular control mechanisms. Higher pressure gradient of LVOT obstruction in HCM patients without HTN as shown in our study might also related to higher prevalence of syncope and sudden death. In addition, more and more evidence has shown that disturbance of reflex control of the vascular system is a common abnormality in HCM patients. This abnormality may lead to abrupt and inappropriate vasodilatation, causing hypotension and consequently recurrent syncope and sudden death^18^. However, it has been shown that higher baseline blood pressure in hypertensive patients provides individuals a greater blood pressure “reserve” for maintenance of consciousness at onset of syncope^20^. Accordingly, it seems rational that the elevated basal blood pressure in HCM patients with HTN counteracts the drop in blood pressure caused by abnormal reflex control of vasculature, thereby reducing the incidence of syncope and sudden death.

The comparison of 5-year survival rate between two groups showed a trend toward poorer prognosis in HCM patients with HTN, although the results were not statistically significant due to insufficient sample size and follow-up time. The leading causes of death in HCM patients are heart failure and sudden cardiac death^21^. However, our study showed that the prevalence of syncope and sudden death was lower in HCM patients with HTN who had a trend toward high mortality, suggesting that the constituent ratio of cause of death in HCM patients with HTN differs from that of patients with HCM alone. As shown in our study, the HTN-induced target organ damage such as cerebral ischemia and chronic renal failure was more pronounced in HCM patients with HTN. HCM patients with HTN were also older at diagnosis and therefore more likely to have other organ dysfunction, such as hyperlipidemia and chronic obstructive pulmonary disease. It has also been shown that patients with both HCM and hypertension have lower myocardial strain than patients with HCM alone, suggesting greater impairment of left ventricular function, which may be related to worse prognosis^22^.

### Limitations

One limitation of this study is that it was a single-center, retrospective study, with a limited sample size, so findings might not be generalized. Besides, our study is a clinical study which lacks biological mechanisms that describe how HTN play a pathogenic role in HCM patients. Thus, further studies conducted in animal models to elucidate the possible mechanism are needed.

## Conclusion

In our study, we found that the clinical phenotype of HCM patients with HTN differs from that of patients with HCM alone, suggesting that HTN may play a pathogenic role in the pathogenesis of HCM patients with HTN, rather than acting as an auxiliary factor. In subsequent studies, a larger sample of patients’ needs to be included for prospective studies. In addition, genome-wide sequencing of HCM patients with HTN may provide useful information regarding their etiology.

## Data Availability

The datasets collected during the study are available from the corresponding author on reasonable request.
